# Supplementary figures and images for: Constitutive 5-HT2C receptor knock-out facilitates fear extinction through altered activity of a dorsal raphe-bed nucleus of the stria terminalis pathway
Source: Transl Psychiatry. 2022 Nov 19;12:487. doi: 10.1038/s41398-022-02252-x (PMC9675804; doi:10.1038/s41398-022-02252-x)

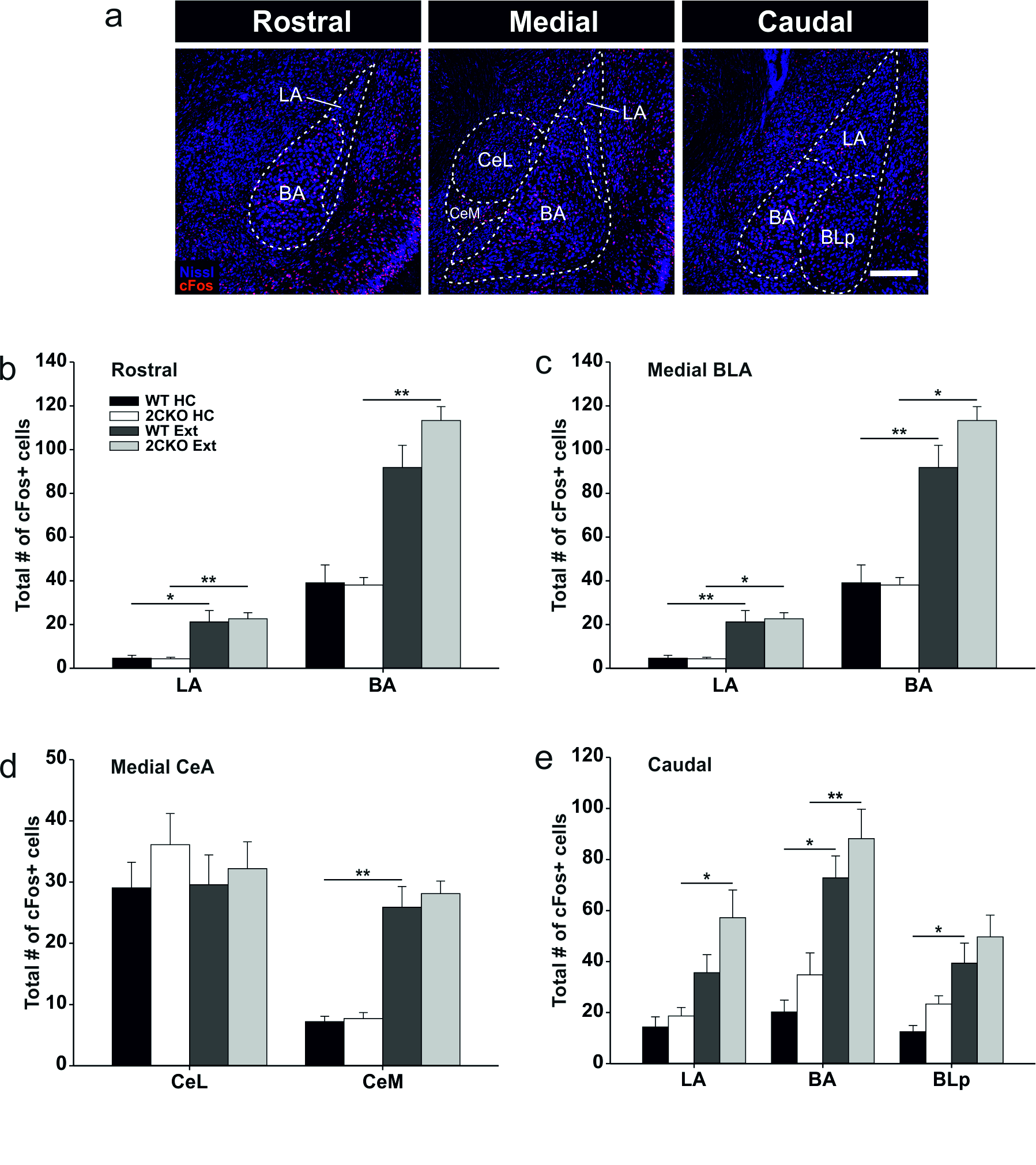

Supplement: Supplementary file 1 — Supplementary Figure 1 [file 41398_2022_2252_MOESM1_ESM.tif]

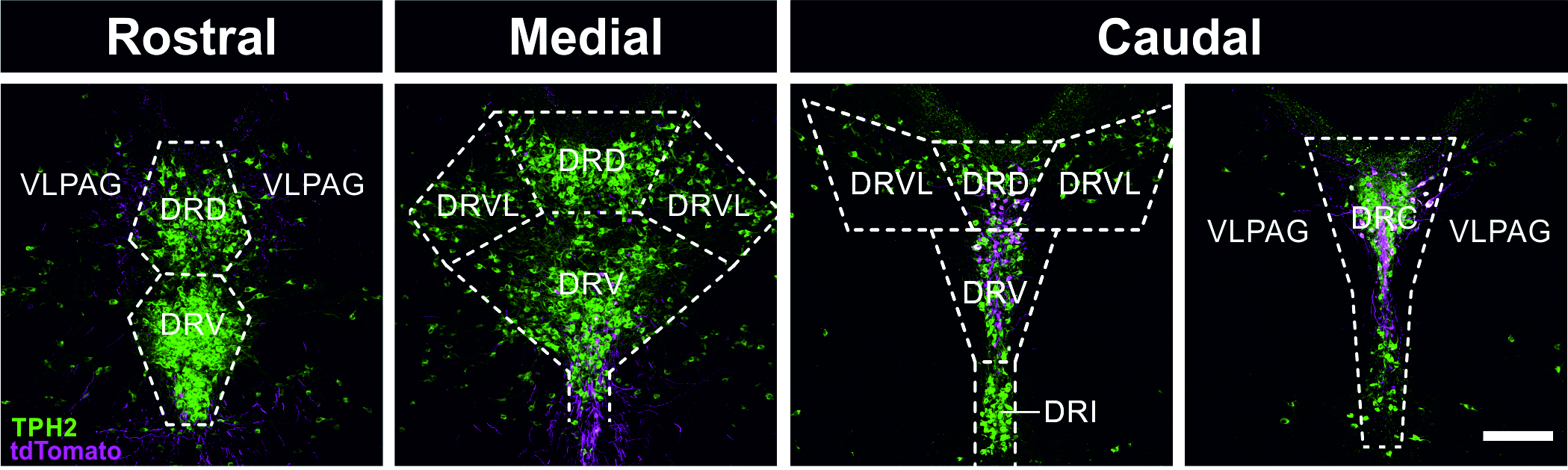

Supplement: Supplementary file 2 — Supplementary Figure 2 [file 41398_2022_2252_MOESM2_ESM.tif]

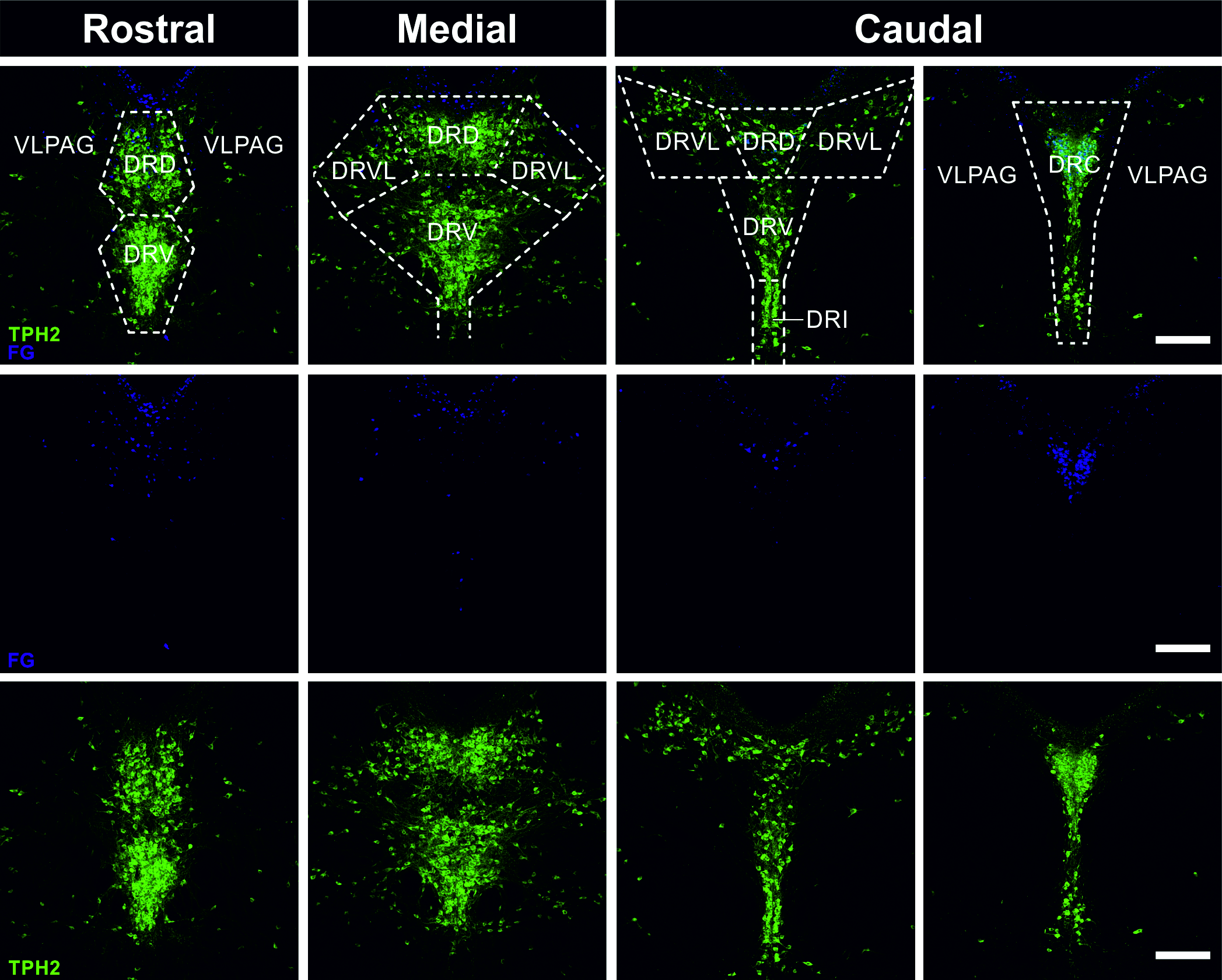

Supplement: Supplementary file 3 — Supplementary Figure 3 [file 41398_2022_2252_MOESM3_ESM.tif]

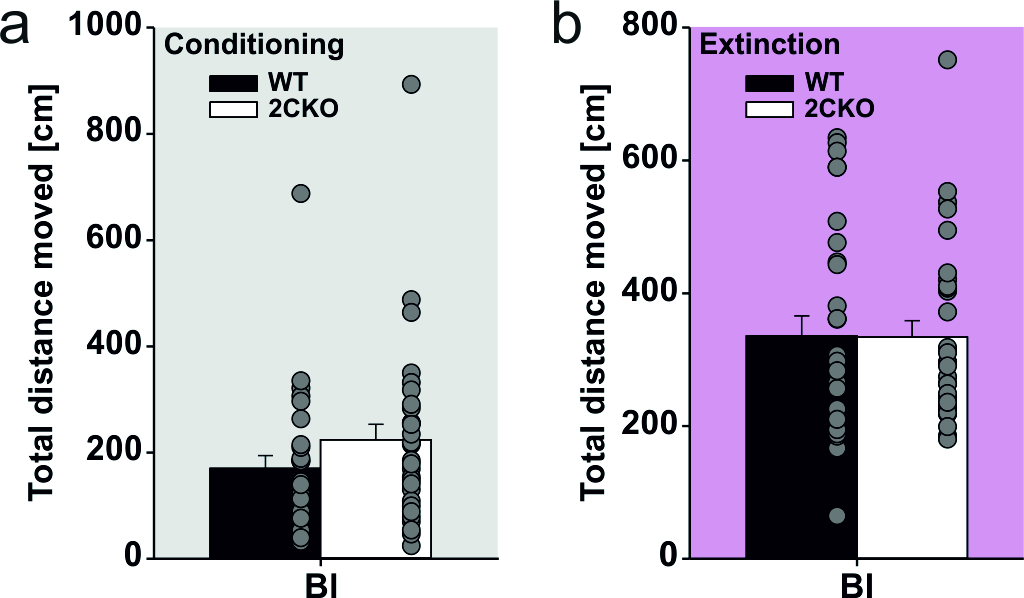

Supplement: Supplementary file 4 — Supplementary Figure 4 [file 41398_2022_2252_MOESM4_ESM.tif]
