# Supplementary material for: Constitutive 5-HT2C receptor knock-out facilitates fear extinction through altered activity of a dorsal raphe-bed nucleus of the stria terminalis pathway
Source: Transl Psychiatry. 2022 Nov 19;12:487. doi: 10.1038/s41398-022-02252-x (PMC9675804; doi:10.1038/s41398-022-02252-x)
Supplement: Supplementary file 5 — Supplementary legend [file 41398_2022_2252_MOESM5_ESM.docx]

**Supplementary Figure 1. Neuronal activity in the amygdala is not altered in 2CKO mice.** (**a**) Representative images of amygdala levels analyzed. Boundaries are outlined by dashed lines. Basolateral amygdala (BLA) encompasses LA = lateral amygdala, BA = basal amygdala and BLp = posterior portion of the basolateral amygdala; central amygdala nucleus (CeA) encompasses CeL = lateral portion and CeM = medial portion. Scale bar = 300 µm. (**b**) cFos quantification in the rostral amygdala. Significant effects LA: Kruskal-Wallis one-way ANOVA on ranks (cFos+): P = < 0.001; pairwise Dunn's test: WT HC vs. WT Ext: P = < 0.05, 2CKO HC vs. 2CKO Ext: P = < 0.01. Significant effects BA: Kruskal-Wallis one-way ANOVA on ranks (cFos+): P = < 0.001; pairwise Dunn's test: 2CKO HC vs. 2CKO Ext: P = < 0.01. (**c**) cFos quantification in the medial BLA. Significant effects LA: Kruskal-Wallis one-way ANOVA on ranks (cFos+): P = < 0.001; pairwise Dunn's test: WT HC vs. WT Ext: P = < 0.01, 2CKO HC vs. 2CKO Ext: P = < 0.05. Significant effects BA: Kruskal-Wallis one-way ANOVA on ranks (cFos+): P = < 0.001; pairwise Dunn's test: WT HC vs. WT Ext: P = < 0.01, 2CKO HC vs. 2CKO Ext: P = < 0.05. (**d**) cFos quantification in the medial CeA. Significant effects CeM: Kruskal-Wallis one-way ANOVA on ranks (cFos+): P = < 0.001; pairwise Dunn's test: WT HC vs. WT Ext: P = < 0.01, 2CKO HC vs. 2CKO Ext: P = < 0.05. (**e**) cFos quantification in the caudal amygdala. Significant effects LA: Kruskal-Wallis one-way ANOVA on ranks (cFos+): P = < 0.001; pairwise Dunn's test: 2CKO HC vs. 2CKO Ext: P = < 0.05. Significant effects BA: Kruskal-Wallis one-way ANOVA on ranks (cFos+): P = < 0.001; pairwise Dunn's test: WT HC vs. WT Ext: P = < 0.01, 2CKO HC vs. 2CKO Ext: P = < 0.05. Significant effects BLp: Kruskal-Wallis one-way ANOVA on ranks (cFos+): P = < 0.001; pairwise Dunn's test: WT HC vs. WT Ext: P = < 0.05. For all graphs (b–e), HC: WT mice (n = 8), 2CKO mice (n = 7); Ext: WT mice (n = 7), 2CKO mice (n = 7). Data are shown as means ± SEM. *P < 0.05, **P < 0.01.

**Supplementary Figure 2. tdTomato expression throughout the DRN.** Representative immuno-stained DRN sections of an ePet1-Cre mouse indicate selective tdTomato (magenta) expression in TPH2+ 5-HT cells (green) in the caudal level of the DRN. The pattern of TPH2+ 5-HT cells (green) was used to define the respective level. Boundaries are outlined by dashed lines. DRD = dorsal raphe nucleus, dorsal part; DRV = dorsal raphe nucleus, ventral part; DRI = dorsal raphe nucleus, interfascicular part; DRC = dorsal raphe nucleus, caudal part; DRVL = dorsal raphe nucleus, ventrolateral part; VLPAG = ventrolateral periaqueductal gray. Scale bar = 200 µm.

**Supplementary Figure 3. Distribution of retrogradely labeled FG cells in the DRN.** Representative immuno-stained DRN sections of a WT mouse injected with 1 % FG into the BNSTad. TPH2+ 5-HT cells (green) in the caudal DRC subregion are densely labeled with FG (blue). Other DRN levels analyzed contain only a few FG-labeled TPH2+ 5-HT cells. The pattern of TPH2+ 5-HT cells (green) was used to define the respective level. Boundaries are outlined by dashed lines. DRD = dorsal raphe nucleus, dorsal part; DRV = dorsal raphe nucleus, ventral part; DRI = dorsal raphe nucleus, interfascicular part; DRC = dorsal raphe nucleus, caudal part; DRVL = dorsal raphe nucleus, ventrolateral part; VLPAG = ventrolateral periaqueductal gray. Scale bars = 200 µm.

**Supplementary Figure 4. Locomotor activity is not altered in 2CKO mice in the fear conditioning and extinction paradigm.** (**a**) Total distance moved during the baseline (Bl) period of the conditioning session (day 2) was similar in both genotypes. (**b**) Total distance moved during the baseline (Bl) period of the extinction session (day 3) was similar in both genotypes. WT mice (n = 29), 2CKO mice (n = 30). Data are shown as means ± SEM.
